# Enterprise ownership, workplace culture, and smoking cessation difficulty in China: A cross-sectional data analysis

**DOI:** 10.18332/tid/226285

**Published:** 2026-08-07

**Authors:** Gang Wang, Kecheng Du

**Affiliations:** 1 Merchants’ Guild Economics and Cultural Intelligent Computing Laboratory, Institute of Modern Communication Research, Ningbo University, Ningbo, China; 2 School of Journalism and Communication, Wuhan University, Wuhan, China

**Keywords:** workplace, smoking cessation, secondhand smoke, enterprise ownership, China

## Abstract

**INTRODUCTION:**

Workplaces are important settings for smoke-free protection and cessation support, but enterprise ownership may shape quit attempts. This cross-sectional data analysis examined whether employees of state-owned enterprises experience greater smoking cessation difficulty than employees of private enterprises.

**METHODS:**

We analyzed cross-sectional self-reported data from an anonymous online questionnaire administered in October 2025. The questionnaire link was distributed through employee WeChat groups in six enterprises in Zhejiang Province, China (three state-owned and three private). Eligible participants were full-time employees aged ≥18 years who had worked in the enterprise for at least six months. The analytic sample comprised 989 employees. Outcomes were workplace cessation culture, indoor workplace secondhand smoke exposure in the past 30 days, quit intention within six months, and past-year quit attempt among current smokers. Ownership-group differences were assessed descriptively and using multivariable linear regression and logistic generalized estimating equations.

**RESULTS:**

The sample included 483 state-owned and 506 private enterprise employees. Current smoking was more common in state-owned enterprises (34.8% vs 27.5%, p=0.046). State-owned enterprise employees reported lower cessation culture (3.33 vs 3.64, p<0.001), less cessation support (2.5 vs 3.6, p<0.001), stronger pro-smoking norms (2.90 vs 2.49, p<0.001), and more indoor workplace secondhand smoke exposure (42.0% vs 25.5%, p<0.001). Among current smokers, quit intention (36.9% vs 52.5%, p=0.009) and past-year quit attempts (29.8% vs 57.6%, p<0.001) were less common in state-owned enterprises. After adjustment, private enterprise employment was associated with higher odds of quit intention (adjusted odds ratio, AOR=1.51; 95% CI: 1.05–2.19) and past-year quit attempt (AOR=2.82; 95% CI: 1.65–4.84).

**CONCLUSIONS:**

State-owned enterprise employees experienced greater cessation difficulty than private enterprise employees. Higher secondhand smoke exposure, stronger pro-smoking norms, weaker cessation support, and lower leadership commitment may contribute to this ownership-related difference.

## INTRODUCTION

Tobacco use remains a major preventable cause of morbidity and mortality worldwide^1^. The WHO Framework Convention on Tobacco Control (WHO FCTC) identifies protection from exposure to tobacco smoke as a core tobacco-control obligation, and Article 8 implementation guidelines emphasize that effective protection requires comprehensive smoke-free indoor environments rather than partial or voluntary restrictions^2,3^. Secondhand smoke exposure has no risk-free level and is causally linked to cardiovascular disease, respiratory disease, lung cancer, and premature death^4,5^. Workplaces are therefore a critical setting for smoke-free protection and cessation-oriented health promotion, because employees spend substantial time at work and are influenced by workplace rules, leadership behavior, coworker norms, and the availability of support services.

The Chinese tobacco-control context makes workplace action especially important. China has a large population of smokers and a substantial burden of tobacco-attributable disease, with pronounced sex differences in smoking patterns and tobacco-related mortality^6-8^. Although national commitments and local smoke-free regulations have expanded, the scope and comprehensiveness of smoke-free laws remain uneven across Chinese cities and venue types^9^. Indoor smoking may persist where rules are incomplete, enforcement is weak, managers tolerate smoking, or cigarettes are embedded in workplace social exchange, hospitality, and business entertainment. Evidence from other settings indicates that comprehensive smoke-free workplace policies can reduce secondhand smoke exposure and may contribute to lower smoking prevalence and increased quitting^10-12^. In parallel, workplace cessation interventions and health-system-supported cessation services can improve cessation outcomes when employees receive concrete assistance, counselling, referral, and social support^13,14^.

Formal policy alone may not be sufficient. Social-contextual models of tobacco use emphasize that smoking behavior is sustained by organizational culture, job conditions, peer practices, and social norms, particularly among working populations whose daily routines and social identities are shaped by the workplace^15^. In China, cigarette sharing and gifting can function as social currency, making tobacco refusal and quit attempts dependent not only on individual motivation but also on the acceptability of declining cigarettes, management role-modeling, and the wider climate around smoking^16^. A workplace with visible smoke-free rules but permissive social norms may still leave employees exposed to secondhand smoke and may fail to normalize quitting.

Enterprise ownership may influence the organizational setting in which smoking norms and tobacco-control practices operate: studies of Chinese firms have identified ownership-related differences in organizational learning culture, with privately owned enterprises reporting a stronger learning atmosphere than state-owned enterprises^17^. Earlier urban evidence also found that employment in a state-owned work unit was associated with a higher likelihood of smoking after adjustment for socioeconomic characteristics^18^. At the workplace-policy level, a large survey spanning companies of different ownership types found that comprehensive smoke-free policies were associated with lower secondhand smoke exposure and current smoking, although the association with quit intention was weak^19^. Together with evidence that cigarette sharing and gifting can impede cessation in China^16^, these findings make enterprise ownership a plausible organizational context for smoking cessation, while also showing that direct comparative evidence on cessation support and quit attempts remains limited. This study uses an employee-level dataset to examine this ownership pathway in Zhejiang Province, China. The aims were to compare state-owned and private enterprises on workplace smoking cessation culture, indoor workplace secondhand smoke exposure, quit intention, and past-year quit attempts, and to examine how smoke-free policy implementation, cessation support, leadership commitment, and pro-smoking social norms help explain the ownership pattern.

## METHODS

### Study design and setting

This cross-sectional, company-based study used employee survey data from six enterprises in Zhejiang Province, China. Data were collected during a one-month survey in October 2025. Employees were clustered within three state-owned enterprises and three private enterprises. State-owned enterprises were defined as enterprises under majority state ownership or effective state control; private enterprises were domestically owned enterprises without state controlling ownership. Tobacco manufacturers, tobacco sellers, and other tobacco-related enterprises were excluded from the sampling frame.

The prespecified analytic framework compared employees by enterprise ownership and examined associations of ownership, workplace tobacco-control conditions, and smoking-related social norms with cessation culture, secondhand smoke exposure, quit intention, and quit attempts.

### Participants and data collection

Eligible participants were full-time employees aged ≥18 years who had worked in the participating enterprise for at least six months. During the survey period, a link to an anonymous, self-administered online questionnaire was posted in employee WeChat groups. The questionnaire landing page explained the study purpose, the voluntary and anonymous nature of participation, and the eligibility criteria; respondents provided electronic informed consent before proceeding. All variables were self-reported. Of 1323 submitted questionnaires, 334 were excluded because respondents were younger than 18 years, were not full-time employees, had worked in the enterprise for less than six months, or did not meet the prespecified questionnaire completeness and logic requirements. The final analytic sample comprised 989 employees.

The three state-owned enterprises contributed 160, 161, and 162 employees, respectively, for a total of 483. The three private enterprises contributed 168, 169, and 169 employees, respectively, for a total of 506.

### Outcomes

The primary outcome was perceived workplace smoking cessation culture. It was calculated as the mean of eight items each rated on five-point Likert scale (1–5), covering encouragement to quit, coworker and management support, acceptability of refusing cigarettes, availability of cessation information, smoke-free norms, fairness of enforcement, and support for quit attempts. The resulting score ranged from 1 to 5, with higher scores indicating a more supportive cessation culture.

Three binary secondary outcomes were assessed. Any indoor workplace secondhand smoke exposure was coded as ‘yes’ if the respondent reported exposure at least once inside the workplace during the past 30 days and ‘no’ otherwise. Quit intention was coded as ‘yes’ if a current smoker intended to quit within the next six months and ‘no’ otherwise. Past-year quit attempt was coded as ‘yes’ if a current smoker reported at least one quit attempt during the previous 12 months and ‘no’ otherwise. Analyses of quit intention and past-year quit attempts were therefore restricted to current smokers.

### Exposure, workplace variables and potential confounders

The main exposure was enterprise ownership, coded as private enterprise or state-owned enterprise, with state-owned enterprise used as the reference category in regression models. Smoke-free policy implementation was the sum of six binary items (0=no, 1=yes) covering a written policy, comprehensive indoor coverage, no-smoking signs, enforcement, correction or penalty for violations, and application of the policy to visitors and clients. Scores ranged from 0 to 6; higher scores indicated more comprehensive implementation. Workplace cessation support was the sum of seven binary items covering cessation education level, referral, counselling, digital resources, peer support, financial incentives, and flexibility in working time for cessation activities. Scores ranged from 0 to 7; higher scores indicated more available support.

Pro-smoking social norms were measured as the mean of six items rated from 1 (strongly disagree) to 5 (strongly agree), with higher scores indicating greater acceptance of smoking, cigarette offering, and smoking during work-related social interaction. Perceived leadership commitment to tobacco control was the mean of four items rated from 1 (strongly disagree) to 5 (strongly agree), with higher scores indicating stronger managerial support for smoke-free rules and cessation. Three additional binary workplace indicators were used in descriptive comparisons: no-smoking signs in all main indoor locations (yes, no), a designated indoor smoking area (yes, no), and any observed evidence of indoor smoking (yes, no).

Employee-level variables were defined as follows: age; sex (female or male), marital status (married or cohabiting vs other); education level (college or higher vs lower than college), monthly income (<5000, 5000–7999, or ≥8000 RMB); occupational position (frontline/production, technical/administrative, or managerial), employment duration (years), weekly working hours, business entertainment (at least weekly vs less often), work stress (high vs not high, according to the prespecified questionnaire cutoff), and household smoking exposure (yes, no). Smoking status was categorized as current smoker, former smoker, or never smoker. Current smokers reported smoking cigarettes at the time of the survey, either daily or occasionally, former smokers reported previous but not current smoking, never smokers reported never smoking.

Age, sex, marital status, education level, income, occupational position, employment duration, weekly working hours, business entertainment, work stress, and household smoking exposure were specified *a priori* as potential confounding factors because they could be associated with both enterprise ownership and smoking-related outcomes. Smoking status was additionally treated as a potential confounder in the full-sample models but was not included in models restricted to current smokers.

### Statistical analysis

Continuous variables were summarized using means and standard deviations (SDs), except employment duration, which was summarized using the median and interquartile range (IQR). Categorical variables were summarized using frequencies and percentages. State-owned and private enterprise employees were compared using independent-samples t-tests for continuous variables summarized by means, the Wilcoxon rank-sum test for employment duration, and Pearson chi-squared tests for categorical variables. These descriptive comparisons included current smoking, business entertainment, pro-smoking social norms, no-smoking signs, designated indoor smoking areas, evidence of indoor smoking, secondhand smoke exposure, cessation support, leadership commitment, smoke-free policy implementation, cessation culture, quit intention, and past-year quit attempts.

Two fully adjusted models were estimated in the complete employee sample (N=989). First, workplace cessation culture was analyzed using multivariable linear regression with robust standard errors clustered at the enterprise level. Second, any indoor workplace secondhand smoke exposure was analyzed using a population-averaged logistic generalized estimating equation model with a logit link, binomial distribution, enterprise as the clustering unit, and an exchangeable working structure to account for within-enterprise dependence. Both models included enterprise ownership, smoke-free policy implementation, workplace cessation support, pro-smoking social norms, leadership commitment, smoking status, age, sex, marital status, education level, income, occupational position, employment duration, weekly working hours, business entertainment, work stress, and household smoking exposure.

Among current smokers (n=307), two separate population-averaged logistic generalized estimating equation models were estimated for: 1) intention to quit within six months; and 2) at least one past-year quit attempt. Both models used a logit link, binomial distribution, enterprise as the clustering unit, and an exchangeable working structure. Each model included enterprise ownership, workplace cessation culture, workplace secondhand smoke exposure, smoke-free policy implementation, workplace cessation support, pro-smoking social norms, leadership commitment, business entertainment, work stress, age, sex, marital status, education level, income, occupational position, employment duration, weekly working hours, and household smoking exposure. Smoking status was not entered because all participants in these models were current smokers.

Linear-model associations are reported as unstandardized regression coefficients (B) with 95% confidence intervals (CIs). Logistic-model associations are reported as adjusted odds ratios (AORs) with 95% CIs. All tests were two-sided, and p<0.05 was considered statistically significant. Analyses were conducted using Stata version 19.0. The questionnaire landing page described the study purpose and the voluntary and anonymous nature of participation, and all participants provided electronic informed consent before proceeding.

## RESULTS

### Sample characteristics

A total of 989 employees from six enterprises were included in the analytic sample, comprising 483 employees from state-owned enterprises and 506 employees from private enterprises. The overall mean age was 37.7 years (SD=8.2), 42.4% of respondents were women, and 44.1% were married or cohabiting. Compared with private enterprise employees, state-owned enterprise employees had a higher mean age (39.2 [SD=8.0] vs 36.2 [SD=8.1] years; p<0.001), longer median employment duration (7 [IQR: 5–11] vs 4 [IQR: 2–6] years; p<0.001), and a higher prevalence of business entertainment at least weekly (20.1% vs 11.9%; p<0.001), whereas mean weekly working hours were lower (44.1 [SD=5.7] vs 45.7 [SD=6.3] hours; p<0.001). The proportion who were married or cohabiting was 47.6% in state-owned enterprises and 40.7% in private enterprises (p=0.034). Descriptive characteristics by enterprise ownership are shown in Table 1.

**Table 1:** Characteristics of employees by enterprise ownership, Zhejiang Province, China, October 2025 (N=989)

*Characteristics*	*Overall* *(N=989)* *n (%)*	*State-owned* *(N=483)* *n (%)*	*Private* *(N=506)* *n (%)*	*p*
**Age** (years), mean (SD)	37.7 (8.2)	39.2 (8.0)	36.2 (8.1)	<0.001
**Female**	419 (42.4)	204 (42.2)	215 (42.5)	0.987
**Married or cohabiting**	436 (44.1)	230 (47.6)	206 (40.7)	0.034
**College education or higher**	648 (65.5)	308 (63.8)	340 (67.2)	0.286
**Monthly income** (RMB)				0.261
<5000	126 (12.7)	59 (12.2)	67 (13.2)	na
5000–7999	400 (40.4)	208 (43.1)	192 (37.9)	na
≥8000	463 (46.8)	216 (44.7)	247 (48.8)	na
**Occupation**				0.964
Frontline/production	481 (48.6)	234 (48.4)	247 (48.8)	na
Technical/administrative	323 (32.7)	157 (32.5)	166 (32.8)	na
Managerial	185 (18.7)	92 (19.0)	93 (18.4)	na
**Employment duration** (years), median (IQR)	5 (3–9)	7 (5–11)	4 (2–6)	<0.001
**Weekly working hours,** mean (SD)	44.9 (6.1)	44.1 (5.7)	45.7 (6.3)	<0.001
**Business entertainment at least weekly**	157 (15.9)	97 (20.1)	60 (11.9)	<0.001
**High work stress**	303 (30.6)	141 (29.2)	162 (32.0)	0.371
**Household smoking exposure**	336 (34.0)	173 (35.8)	163 (32.2)	0.259

Percentages may not sum to 100% because of rounding. P-values are from Pearson chi-squared tests, independent-samples t-tests, or the Wilcoxon rank-sum test, as appropriate. na: not applicable. IQR: interquartile range. RMB: 1000 Chinese Renminbi about US$150.

There were no statistically significant differences between ownership groups in sex, education level, monthly income, occupational position, high work stress, or household smoking exposure (all p>0.05).

### Smoking-related and workplace tobacco-control characteristics

Current smoking was reported by 307 employees (31.0% of the full sample). Current smoking was more common in state-owned enterprises than in private enterprises (34.8% vs 27.5%; p=0.046). Former smoking was reported by 9.0% of respondents, and 60.0% reported never smoking.

The mean workplace smoking cessation culture score was 3.49 (SD=0.51) in the full sample, 3.33 (SD=0.48) among state-owned enterprise employees, and 3.64 (SD=0.49) among private enterprise employees (private minus state-owned, mean difference=0.31; 95% CI: 0.25–0.37; p<0.001). Compared with state-owned enterprise employees, private enterprise employees had a higher mean cessation support index (3.6 [SD=1.3] vs 2.5 [SD=1.2]; p<0.001), higher mean leadership commitment score (3.87 [SD=0.64] vs 3.40 [SD=0.65]; p<0.001), and lower mean pro-smoking social norms score (2.49 [SD=0.69] vs 2.90 [SD=0.68]; p<0.001). No-smoking signs in all main indoor locations were reported by 77.3% of private enterprise employees and 72.3% of state-owned enterprise employees (p=0.081). Designated indoor smoking areas (18.4% vs 6.7%; p<0.001) and evidence of indoor smoking (17.4% vs 8.5%; p<0.001) were more common in state-owned enterprises than in private enterprises (Table 2).

**Table 2:** Smoking-related and workplace tobacco-control characteristics by enterprise ownership, Zhejiang Province, China, October 2025 (N=989)

*Characteristics*	*Overall* *(N=989)* *n (%)*	*State-owned* *(N=483)* *n (%)*	*Private* *(N=506)* *n (%)*	*p*
**Smoking status**				0.046
Current smoker	307 (31.0)	168 (34.8)	139 (27.5)	na
Former smoker	89 (9.0)	41 (8.5)	48 (9.5)	na
Never smoker	593 (60.0)	274 (56.7)	319 (63.0)	na
	***Mean (SD)***	***Mean (SD)***	***Mean (SD)***	
Workplace cessation culture	3.49 (0.51)	3.33 (0.48)	3.64 (0.49)	<0.001
Smoke-free policy implementation index	4.3 (1.0)	4.1 (1.1)	4.5 (1.0)	<0.001
Workplace cessation support index	3.1 (1.3)	2.5 (1.2)	3.6 (1.3)	<0.001
Leadership commitment	3.64 (0.69)	3.40 (0.65)	3.87 (0.64)	<0.001
Pro-smoking social norms	2.69 (0.71)	2.90 (0.68)	2.49 (0.69)	<0.001
	***n (%)***	***n (%)***	***n (%)***	
No-smoking signs in all main indoor locations	740 (74.8)	349 (72.3)	391 (77.3)	0.081
Designated indoor smoking area reported	123 (12.4)	89 (18.4)	34 (6.7)	<0.001
Evidence of indoor smoking observed	127 (12.8)	84 (17.4)	43 (8.5)	<0.001
Any indoor workplace SHS exposure in past 30 days	332 (33.6)	203 (42.0)	129 (25.5)	<0.001
Intention to quit within six months among current smokers	135 (44.0)	62 (36.9)	73 (52.5)	0.009
Past-year quit attempt among current smokers	130 (42.3)	50 (29.8)	80 (57.6)	<0.001

P-values are from Pearson chi-squared tests or independent-samples t-tests, as appropriate. Workplace cessation culture, leadership commitment, and pro-smoking social norms scores range from 1 to 5; the smoke-free policy implementation index ranges from 0 to 6; and the workplace cessation support index ranges from 0 to 7. Higher values indicate a more supportive condition except for pro-smoking social norms, for which higher values indicate greater acceptance of smoking. an: not applicable. SHS: secondhand smoke.

Any indoor workplace secondhand smoke exposure in the past 30 days was reported by 33.6% of all respondents. Exposure was reported by 42.0% of state-owned enterprise employees and 25.5% of private enterprise employees (p<0.001). Among current smokers, intention to quit within six months was reported by 36.9% of state-owned enterprise employees and 52.5% of private enterprise employees (p=0.009), and at least one past-year quit attempt was reported by 29.8% and 57.6%, respectively (p<0.001).

### Models for cessation culture and secondhand smoke exposure

In the multivariable linear regression model, private enterprise ownership was not statistically associated with workplace cessation culture after adjustment for employee covariates and workplace tobacco-control factors (B= -0.02; 95% CI: -0.14–0.09; p=0.679).

In the same model, each additional point in the smoke-free policy implementation index was associated with a 0.06-point higher culture score (B=0.06; 95% CI: 0.04–0.07; p<0.001), and each additional point in the cessation support index was associated with a 0.10-point higher culture score (B=0.10; 95% CI: 0.09–0.11; p<0.001). Each one-point increase in leadership commitment was associated with a 0.29-point higher culture score (B=0.29; 95% CI: 0.28–0.31; p<0.001), whereas each one-point increase in pro-smoking social norms was associated with a 0.16-point lower culture score (B= -0.16; 95% CI: -0.19 – -0.12; p<0.001) (Table 3).

**Table 3:** Multivariable models for workplace cessation culture and workplace secondhand smoke exposure, Zhejiang Province, China, October 2025 (N=989)

*Variables*	*Cessation culture* *B (95% CI)*	*p*	*Workplace SHS exposure* *AOR (95% CI)*	*p*
Private enterprise vs state-owned enterprise (ref.)	-0.02 (-0.14–0.09)	0.679	0.64 (0.44–0.91)	0.014
Smoke-free policy implementation, per item	0.06 (0.04–0.07)	<0.001	0.94 (0.83–1.06)	0.294
Workplace cessation support, per item	0.10 (0.09–0.11)	<0.001	1.01 (0.89–1.14)	0.878
Pro-smoking social norms, per point	-0.16 (-0.19 – -0.12)	<0.001	1.40 (1.18–1.67)	<0.001
Leadership commitment, per point	0.29 (0.28–0.31)	<0.001	0.89 (0.75–1.05)	0.176
Current smoker vs never smoker (ref.)	-0.04 (-0.07 – -0.00)	0.034	1.97 (1.69–2.30)	<0.001
Former smoker vs never smoker (ref.)	0.05 (-0.01–0.12)	0.124	0.77 (0.40–1.49)	0.434

For workplace cessation culture, multivariable linear regression was fitted with robust standard errors clustered at the enterprise level. For workplace secondhand smoke exposure, a population-averaged logistic generalized estimating equation model was fitted with an exchangeable working structure and enterprise as the clustering unit. Both models included enterprise ownership, smoking status, age, sex, marital status, education level, income, occupational position, employment duration, weekly working hours, business entertainment, work stress, household smoking exposure, smoke-free policy implementation, workplace cessation support, pro-smoking social norms, and leadership commitment. AOR: adjusted odds ratio. B: unstandardized regression coefficient.

In the population-averaged logistic generalized estimating equation (GEE) model for workplace secondhand smoke exposure, private enterprise ownership was associated with lower odds of exposure (AOR=0.64; 95% CI: 0.44–0.91; p=0.014). Higher pro-smoking social norms were associated with higher odds of exposure (AOR=1.40; 95% CI: 1.18–1.67; p<0.001), and current smokers had higher odds of exposure than never smokers (AOR=1.97; 95% CI: 1.69–2.30; p<0.001).

### Quitting-related outcomes among current smokers

In the fully adjusted population-averaged logistic GEE models among 307 current smokers, private enterprise employment was associated with higher odds of intention to quit within six months (AOR=1.51; 95% CI: 1.05–2.19; p=0.027) and of at least one past-year quit attempt (AOR=2.82; 95% CI: 1.65–4.84; p<0.001) compared with state-owned enterprise employment.

Workplace cessation support was associated with higher odds of quit intention (AOR=1.23; 95% CI: 1.05–1.43; p=0.009). Leadership commitment was associated with higher odds of quit intention (AOR=1.46; 95% CI: 1.04–2.04; p=0.029) and a past-year quit attempt (AOR=1.23; 95% CI: 1.05–1.44; p=0.011). Workplace secondhand smoke exposure was associated with lower odds of quit intention (AOR=0.50; 95% CI: 0.37–0.68; p<0.001). Workplace cessation culture was associated with higher odds of a past-year quit attempt (AOR=1.49; 95% CI: 1.02–2.16; p=0.037).

Smoke-free policy implementation was not statistically associated with quit intention (AOR=1.00; 95% CI: 0.88–1.14; p=0.965) and was associated with lower odds of a past-year quit attempt (AOR=0.79; 95% CI: 0.66–0.95; p=0.011). High work stress was associated with lower odds of quit intention (AOR=0.74; 95% CI: 0.59–0.94; p=0.013) (Table 4).

**Table 4:** Logistic generalized estimating equation models for quitting-related outcomes among current smokers, Zhejiang Province, China, October 2025 (N=307)

*Variables*	*Quit intention within* *six months* *AOR (95% CI)*	*p*	*Past-year quit* *attempt* *AOR (95% CI)*	*p*
Private enterprise vs state-owned enterprise (ref.)	1.51 (1.05–2.19)	0.027	2.82 (1.65–4.84)	<0.001
Workplace cessation culture, per point	1.38 (0.91–2.09)	0.133	1.49 (1.02–2.16)	0.037
Any workplace SHS exposure in past 30 days vs none (ref.)	0.50 (0.37–0.68)	<0.001	0.69 (0.44–1.08)	0.103
Smoke-free policy implementation, per item	1.00 (0.88–1.14)	0.965	0.79 (0.66–0.95)	0.011
Workplace cessation support, per item	1.23 (1.05–1.43)	0.009	0.97 (0.75–1.26)	0.838
Pro-smoking social norms, per point	1.03 (0.71–1.50)	0.862	1.03 (0.78–1.34)	0.852
Leadership commitment, per point	1.46 (1.04–2.04)	0.029	1.23 (1.05–1.44)	0.011
Business entertainment at least weekly vs less often (ref.)	0.93 (0.69–1.25)	0.633	0.73 (0.40–1.34)	0.312
High work stress vs not high (ref.)	0.74 (0.59–0.94)	0.013	1.27 (0.75–2.14)	0.371

Models were adjusted for age, sex, marital status, education level, income, occupational position, employment duration, weekly working hours, household smoking exposure, business entertainment, work stress, and the workplace tobacco-control variables listed in the table. AOR: adjusted odds ratio. SHS: secondhand smoke.

## DISCUSSION

This study identified an ownership-related pattern in workplace tobacco-control conditions and quitting outcomes. Compared with private enterprise employees, state-owned enterprise employees had higher current smoking prevalence and workplace secondhand smoke exposure, more designated indoor smoking areas and evidence of indoor smoking, lower cessation support and leadership commitment, and stronger pro-smoking social norms. Among current smokers, quit intention and past-year quit attempts were less common in state-owned enterprises; after adjustment, private enterprise employment remained associated with higher odds of both outcomes.

The descriptive difference in cessation culture by ownership was attenuated after workplace factors and employee covariates were included, whereas cessation support, leadership commitment, smoke-free policy implementation, and pro-smoking norms remained associated with cessation culture. This pattern is compatible with evidence that Chinese state-owned and privately owned enterprises differ in organizational learning culture^17^. It is also consistent with findings from smaller workplaces in which organizational culture was associated with workplace health climate and smoking status, although not with every smoking outcome^20^.

The greater secondhand smoke exposure and more frequent evidence of indoor smoking reported by state-owned enterprise employees are consistent with evidence that comprehensive smoke-free workplace policies reduce secondhand smoke exposure and smoking and can support cessation^10-12^. A multi-company study in China also found that comprehensive workplace policies were associated with less secondhand smoke exposure and current smoking, but only weakly with quit intention^19^. These comparisons are compatible with the present finding that ownership remained associated with secondhand smoke exposure after adjustment, whereas formal policy implementation alone was not associated with quit intention.

Business entertainment may be one social context underlying ownership differences, although it was not established as a mediator in this analysis. In China, cigarette sharing and gifting can operate as social currency and have been linked to relationship building, status, hospitality, and business exchange^16,18,21^. These norms provide a plausible context for the higher frequency of weekly business entertainment and stronger pro-smoking norms observed in state-owned enterprises. Because the data were cross-sectional, whether business entertainment preceded the quitting-related outcomes cannot be determined.

Leadership commitment was positively associated with cessation culture, quit intention, and past-year quit attempts. Kava et al.^20^ reported that organizational culture was related to workplace health climate and that a more favorable health climate was associated with lower current smoking, but not quit intention. The difference may reflect variation in measurement, workplace size, and national context; nevertheless, both studies distinguish organizational climate from written policy alone.

Workplace cessation support was associated with quit intention, and workplace cessation culture was associated with past-year quit attempts. These results are consistent with reviews showing that counselling, pharmacotherapy, incentives, and other workplace or health-system supports can improve cessation outcomes^13,14^, and with a Finnish public-sector cohort in which employer-provided cessation support was associated with subsequent quitting^22^. A Beijing study also reported associations of environmental and social support with smoking cessation^23^. In the present study, secondhand smoke exposure was inversely associated with quit intention, indicating that exposure control and cessation support represented related but distinct workplace conditions.

Smoke-free policy implementation was not associated with quit intention and was inversely associated with past-year quit attempts in the fully adjusted model. This counterintuitive association should not be interpreted as evidence that stronger policies discourage quitting. Cross-sectional temporality, the small number of enterprise clusters, overlap with culture and support measures, and policy adoption in response to smoking burden could have contributed. The weak association between formal workplace policy and quit intention in a previous Chinese multi-company study also indicates that written policy and quitting motivation may not move in parallel^19^.

High work stress was associated with lower quit intention. A prospective study of Finnish public-sector employees similarly found that smoking cessation was less common in work units characterized by high job strain and low job control^24^. Direct comparison is limited because the present study used a binary self-reported stress measure and assessed quit intention rather than observed cessation.

### Strengths and limitations

This study has several strengths. It used employee-level data from state-owned and private enterprises, applied explicit definitions of ownership and outcomes, assessed multiple components of workplace tobacco control, and used models that accounted for clustering within enterprises. Separating formal policy implementation from cessation support, leadership commitment, and social norms allowed these workplace dimensions to be examined concurrently.

Several limitations should be considered. First, the cross-sectional design precludes causal inference and does not establish the temporal order of ownership-related conditions and quitting outcomes. Second, only six enterprises in one province were included, limiting the generalizability of the findings to other enterprises, industries, and regions. The small number of enterprise clusters also reduces the precision of cluster-adjusted estimates.

Third, all variables were self-reported, which may have introduced information bias and misclassification; measurement of exposures and outcomes from the same questionnaire may also have produced common-method bias. Residual confounding remains possible despite multivariable adjustment. Fourth, the analysis did not include detailed measures of industry-specific rules, local enforcement, collective bargaining, occupational health resources, or regional tobacco-control differences.

Evidence from larger and more geographically diverse samples, longitudinal designs, and multiple data sources would be needed to assess generalizability, reduce common-method bias, and establish temporal ordering.

## CONCLUSIONS

In this study, state-owned enterprises showed weaker cessation support, stronger pro-smoking social norms, higher indoor workplace secondhand smoke exposure, and less favorable quitting-related outcomes than private enterprises. Among current smokers, private enterprise employment remained associated with higher quit intention and past-year quit attempts after adjustment. The results identify enterprise ownership as an organizational context and highlight leadership commitment, cessation support, social norms, indoor smoking conditions, and work stress as correlates of quitting-related outcomes. Causal and mediation pathways cannot be established from these cross-sectional data.

## Data Availability

The data supporting this research are available from the authors on reasonable request.
